# Coordination dynamics in singing with human and artificial partners: the role of visual information

**DOI:** 10.3389/fcogn.2026.1810330

**Published:** 2026-06-15

**Authors:** Rina Nishiyama, Tetsushi Nonaka

**Affiliations:** Graduate School of Human Development and Environment, Kobe University, Kobe, Japan

**Keywords:** anticipatory synchronization, interpersonal coordination, self-other integration, togetherness, visual information

## Abstract

**Introduction:**

This study examined whether coordination dynamics in singing differ when a person sings along with a human partner vs. an artificial partner generated by voice synthesis software (VOCALOID 6). We further investigated how the presence or absence of visual information shapes these coordination patterns.

**Methods:**

Thirteen female participants sang a chorus excerpt of Silent Night in unison with a partner heard through earphones. The task crossed two partner types (a human singer and an artificial VOCALOID 6 singer) with two visual conditions (with and without partner video). Artificial-partner videos were created using an AI video platform (Hedra Character 3) that combined the artificial partner's synthesized voice with a still image of the human partner. Coordination was assessed through cross-correlation and Granger causality analyses of vocal amplitude envelopes, evaluated using linear mixed-effects models.

**Results:**

Singing with a human partner elicited stronger anticipatory synchronization and greater similarity in amplitude envelopes than singing with an artificial partner. With the human partner, participants tended to sing slightly ahead of their partner, indicative of anticipatory coordination. Notably, this tendency was further strengthened when visual information was available.

**Discussion:**

These findings indicate that the temporal variability of the human singing voice, together with the fine-grained bodily movements characteristic of naturalistic vocal production, provides perceptual information that supports anticipation and synchronization in joint singing.

## 1 Introduction

Temporal synchronization and coordination in musical interaction are closely linked to social functioning (Clayton et al., 2020). Musical performance is therefore widely regarded as an inherently social activity, and music is thought to be particularly effective in promoting interpersonal coordination by supporting nonverbal communication, shared experience, and social connectedness (Dotov et al., 2024; Volpe et al., 2016; Welch et al., 2014; Chang et al., 2017; Keller et al., 2017). Successful musical synchronization involves not only perceiving and responding to others' sounds and actions, but also anticipating upcoming events, continuously adjusting timing, and exerting prospective control over one's own actions (Proksch et al., 2022; Keller et al., 2007; Konvalinka et al., 2010; Sebanz et al., 2006). For these reasons, musical interaction has been proposed as an ideal model system for investigating the dynamics of complex coordinated behavior (Proksch et al., 2022; D'Ausilio et al., 2015). More broadly, musical performance provides valuable insights into social engagement (McCallum and McOwan, 2018) and offers a promising framework for advancing research on human–machine and human–robot interaction.

In parallel with these theoretical developments, recent advances in information science and technology have enabled increasingly human-like acoustic synthesis (Umbert et al., 2015; Kühne et al., 2020; Robinson et al., 2022; Wang et al., 2024). Singing voice synthesis technology, in particular, allows machines to generate singing voices that closely resemble those of humans. Since the release of VOCALOID (Yamaha Corporation, Hamamatsu, Japan), a vocal synthesis system based on this technology, its use has expanded rapidly (Kenmochi and Ohshita, 2007). Previous research has shown that sound and voice play a central role in human–robot interaction and has documented advances in the timbre, expressiveness, and coordination capabilities of machine-based musical performance (Sunardi and Perkowski, 2017; Robinson et al., 2022; Wang et al., 2024). Nevertheless, genuinely collaborative musical performance between humans and machines remains difficult to achieve, largely because it requires fine-grained motor coordination and precise temporal synchronization (Wang et al., 2024). More generally, the development of artificial partners capable of supporting seamless, reciprocal collaboration with humans continues to pose a substantial challenge (Dotov et al., 2024; Wang et al., 2024).

Studies of human interaction and coordination have emphasized that interpersonal coordination relies on multimodal bodily processes, including gesture, gaze, bodily orientation, and the prospective interpretation of others' actions (Goodwin, 2000, 2010; Clark, 2005). From this perspective, successful coordination depends not only on reactive synchronization to external events, but also on the anticipatory use of auditory and visual information generated through embodied interaction. Consistent with this view, a growing body of research suggests that bodily movement plays a critical role in communication and expressive coordination during human musical performance (Klein et al., 2022). In addition to auditory information, visual information contributes substantially to interpersonal synchronization (Jakubowski et al., 2020). Studies of musical duet performance, in particular, have shown that performers rely on gaze, body movement, and nonverbal gestures to support prediction and coordination during joint performance (Bishop and Goebl, 2015; Kawase, 2014). Such visual access provides information that is highly beneficial for timing and coordination (Bishop et al., 2019), and bodily movement supports the anticipation of others' actions required for interpersonal synchronization (Keller et al., 2007; Burger et al., 2014). Continuous visual feedback further complements auditory information and facilitates anticipation of others' sounds (Sabharwal et al., 2024). Taken together, these findings suggest that bodily movement is reflected in the sounds spontaneously produced by human performers and can be perceived—even through auditory events alone—as information that supports coordination. The availability of visual information may further enhance this process (Klein et al., 2022). An open question, however, is whether visual information exerts a comparable influence when the interaction partner is artificial rather than human.

Our previous study addressed part of this question in the auditory domain. We demonstrated that, even in the absence of visual information, singing with a human partner elicited stronger anticipatory synchronization and greater similarity in the unfolding dynamics of vocal amplitude envelopes than singing with an artificial partner (Nishiyama and Nonaka, 2024). The result suggested that the temporal variability inherent in the human singing voice facilitates anticipatory synchronization in joint singing. Building on this finding, the present study introduces conditions in which participants could see their singing partner. This extension allows us to examine whether the previously observed differences in coordination persist when visual information—known to affect interpersonal coordination across various musical contexts—is available. Specifically, we ask whether singing with a human partner continues to yield stronger anticipatory synchronization regardless of visual information, or whether the addition of visual information enables interaction with an artificial partner to approach the coordination dynamics observed in human–human singing.

To address these questions, we employed VOCALOID AI implemented in VOCALOID 6 (Yamaha Corporation, Hamamatsu, Japan) to generate artificial singing voices, following Nishiyama and Nonaka (2024). In addition, we used Hedra Character 3 (Hedra Studio; https://www.hedra.com) to generate visually embodied artificial partners whose facial and upper-body movements were synchronized with the synthesized singing voices. As in our previous study, the artificial partner maintained a constant tempo throughout the song, whereas the human partner sang at a self-selected tempo following an initial metronome cue. This contrast was designed to isolate the effect of natural tempo fluctuations—inherent in human singing—on coordination dynamics in joint singing. The key extension in the present study is the inclusion of visual information of bodily movement involved in singing. Although the artificial partner's movements were AI-generated, they nonetheless provided visual cues that could potentially support synchronization. If subtle, human-specific variations exist not only in singing voices but also in bodily movement, it is therefore likely that singing with a human partner would again result in stronger coordination.

To empirically assess coordination dynamics, we applied cross-correlation (CC) and Granger causality (GC) analyses to time series of amplitude envelopes extracted from participants' and partners' singing voices. CC quantifies similarity between two time series across a range of positive and negative time lags, providing information about both temporal alignment and synchrony, particularly at zero lag (Klein et al., 2022). The lag at which the maximum CC occurs further indicates temporal precedence between the two signals. Granger causality (GC), in turn, provides a statistical framework for assessing directional predictive relationships between time series, based on the principle that causes precede effects and improve anticipations of future states (Granger, 1969; Barnett and Seth, 2014). In the context of joint vocal performance, GC can reveal anticipatory dynamics in which one performer's vocal activity anticipates the near-future dynamics of the other. Importantly, GC has been shown to capture such anticipatory structure in coordinated behavior, including situations in which follower actions precede and anticipate leader actions (Chen et al., 2023).

Based on this framework, we formulated the following hypotheses. First, consistent with Nishiyama and Nonaka (2024), we hypothesized that higher maximum CC values and higher zero-lag CC values would be observed when participants sang in unison with a human partner than when they sang with a Vocaloid-generated singing voice, reflecting stronger alignment in vocal dynamics. Second, we hypothesized that the lag at which maximum CC occurred, together with asymmetries in Granger causality directionality, would indicate a greater degree of prospective adjustment during joint singing with a human partner than with an artificial partner. Finally, we hypothesized that all coordination-related measures would be enhanced in the presence of visual information, that is, when participants were able to see their singing partner.

## 2 Methods

### 2.1 Participants

Ethical approval for this study (approval no. 871) was obtained from the Ethics Committee of the Graduate School of Human Development and Environment, Kobe University (Japan). Thirteen female university students participated. All participants reported prior experience singing in group settings (e.g., school choirs). The mean age was 25.2 years (SD = 5.8). A semi-professional female singer was recruited to record the human partner stimuli. No participant reported hearing or motor impairments. Participants received a small honorarium.

### 2.2 Music materials

#### 2.2.1 Audio materials

**Figure 1:**
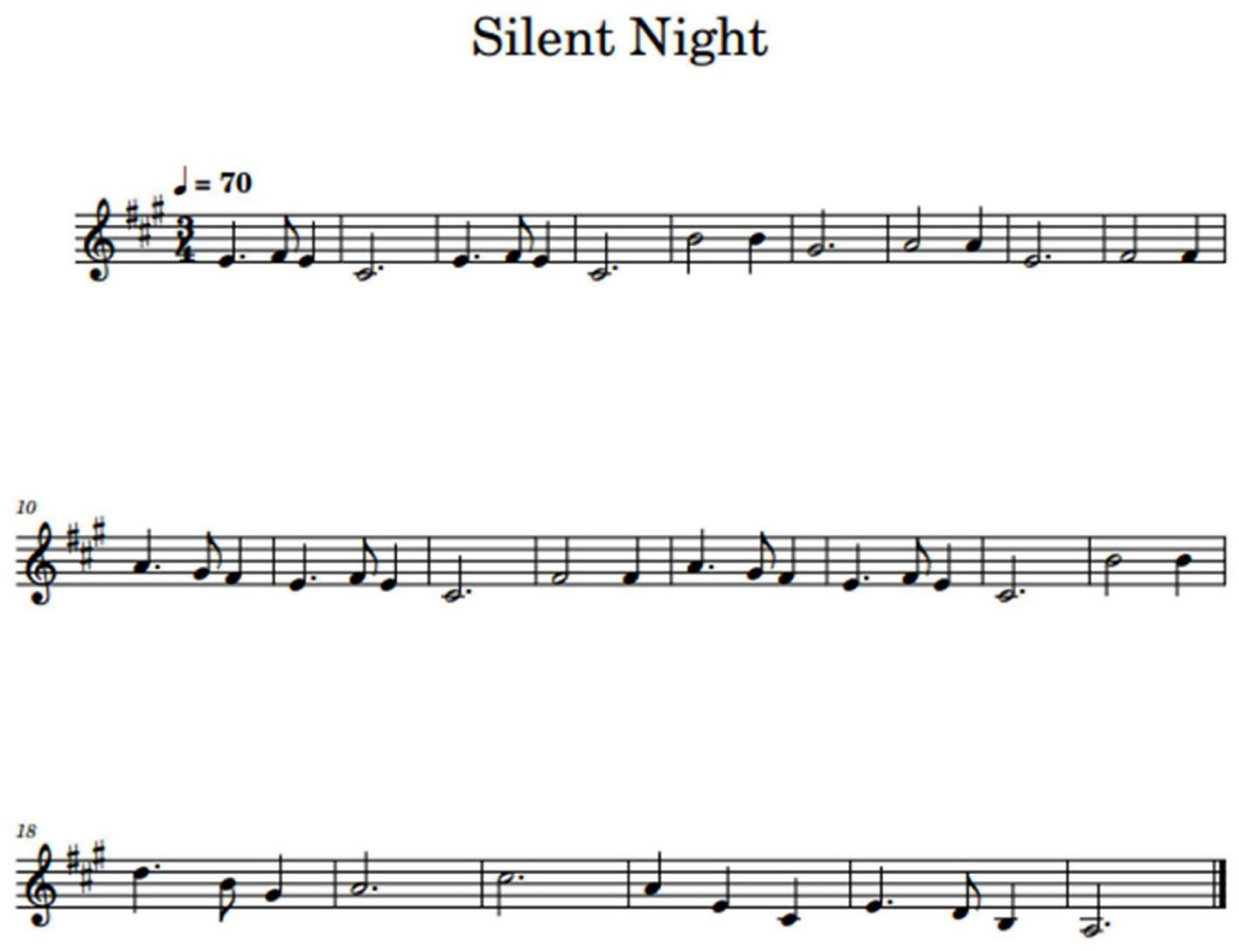
The refrain of “*Silent Night”* (composed by Joseph Mohr, lyrics by Franz Gruber) used in the experiment. Participants sang this excerpt in Japanese.

Participants were asked to sing along in Japanese with an excerpt from *Silent Night* (tempo: 70 BPM, 3/4 time; Figure 1), a hymn with lyrics by Joseph Mohr and music by Franz Gruber. The chorus section of the song was used in the experiment (23 bars; approximately 55 s). As in Nishiyama and Nonaka (2024), two versions of the partner's singing voice were prepared: a human singing voice and an artificial singing voice generated using VOCALOID AI. The artificial partner's singing voice was created using VOCALOID 6 voice synthesis software (voice: HARUKA; audio files are provided in the Supplementary materials). These synthesized recordings were then sent to a semi-professional female singer, who was instructed to reproduce the Vocaloid performance as closely as possible in terms of vocal style and pitch contour. The human singer's voice was recorded using a unidirectional dynamic headset microphone (HYP-190H; Audio-Technica, Japan). Both the human and artificial singing voices were recorded in Cubase LE AI Elements 13 (Steinberg, Hamburg, Germany) as monophonic 16-bit WAV files at a sampling rate of 44.1 kHz (audio files available in the Supplementary materials). Each recording included two measures of metronome clicks prior to the onset of the chorus, which served to indicate the initial tempo. No metronome clicks were present once the chorus began in either partner condition. Consequently, while the artificial Vocaloid singer maintained a constant tempo throughout the chorus, the human singer performed at a self-selected tempo following the initial two-bar metronome cue, resulting in natural tempo fluctuations.

#### 2.2.2 Visual materials

Visual materials consisted of videos depicting participants singing alongside their partners. For the human partner condition, the singer was recorded using a video camera positioned approximately 1.5 m in front of the performer. For the artificial partner condition, singing videos were generated using *Hedra Character 3* (Hedra Studio; https://www.hedra.com), an AI-based platform that transforms still images into dynamic videos (Behforouz and Al Ghaithi, 2025). This system generates videos by combining a still image of a human face (the human partner) with a prepared VOCALOID AI singing voice, automatically synchronizing the character's mouth movements to the input audio via lip-syncing. Among available AI video-generation platforms, *Hedra Character 3* was selected because it produces high-quality videos with controllable facial expressions and accurate lip-syncing (Kitat, 2025). The resulting videos include visually plausible movements and expressions, creating the appearance that the synthesized singing voice is being naturally produced by the depicted performer. To standardize visual information across conditions, we instructed the system to maintain eye contact with the camera while allowing natural upper-body sway during performance. The prompt used to generate the artificial partner videos was as follows:

“*Animate the person in the image to look like they're singing naturally to the provided voice, while maintaining eye contact with the camera. The motion should be smooth and realistic, with subtle but expressive head movements and gentle body swaying to the rhythm of the music. Add natural lip-sync perfectly matching the voice, and focus on the upper body and facial expressions to convey emotion and engagement.”*

In both the human and artificial partner conditions, performers faced forward, and video recordings captured movements from the collarbone upward. Video files for both partner conditions are available in the Supplementary material.

### 2.3 Procedure

**Figure 2:**
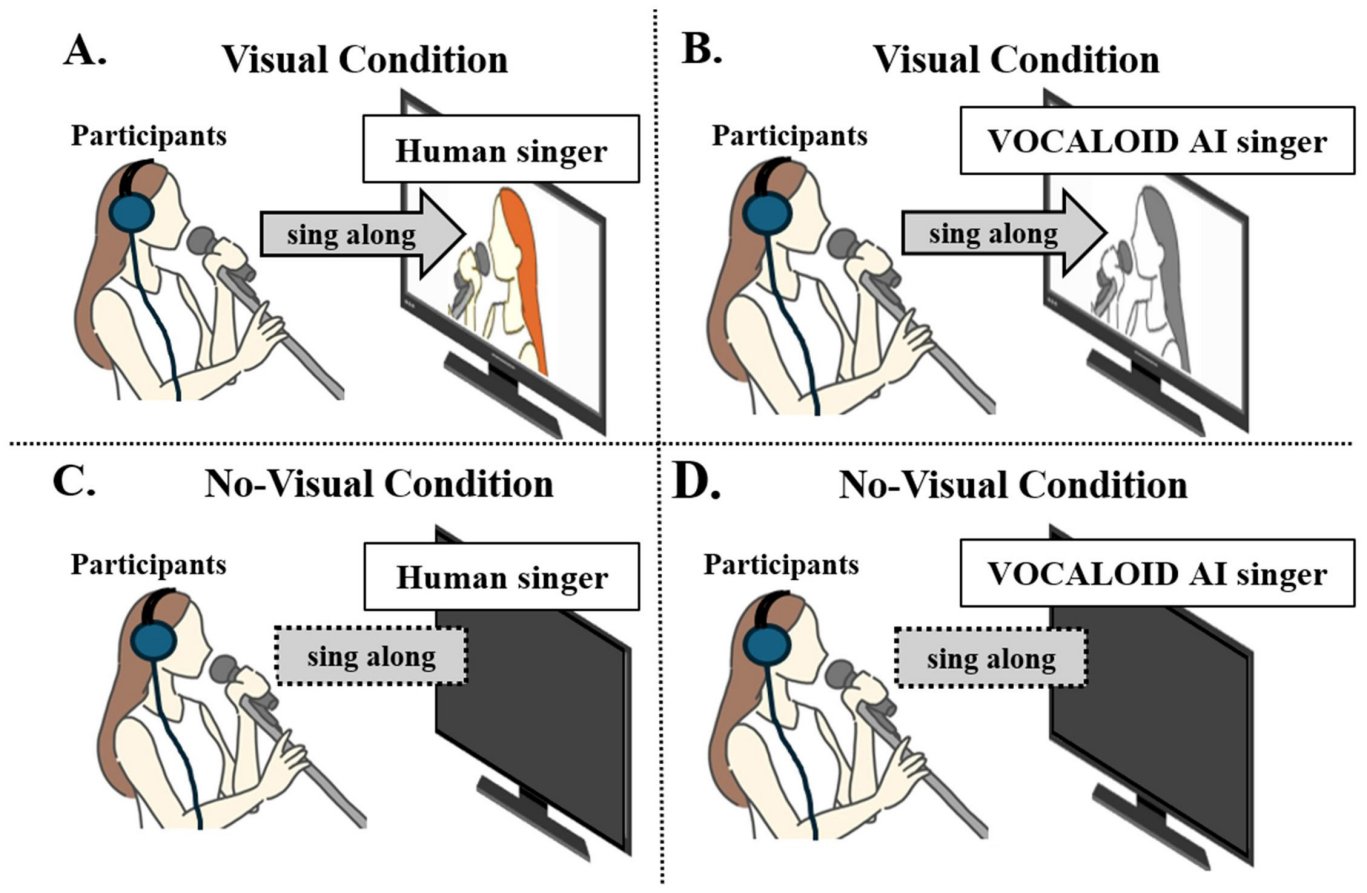
Experimental setup and conditions. **(A)** Human partner condition with visual information, in which participants sang along in unison with a human partner while viewing the partner's singing video. **(B)** Artificial partner condition with visual information, in which participants sang in unison with a Vocaloid-generated singing voice while viewing a video generated from the synthesized voice using an AI video platform (Hedra Character 3). **(C)** Human partner condition without visual information, in which participants sang in unison with a human partner while the screen remained black, with no visual display of the partner. **(D)** Artificial partner condition without visual information, in which participants sang in unison with a Vocaloid-generated singing voice while the screen remained black, with no visual display of the partner.

Participants were informed in advance of the song to be sung in the experiment and were instructed to memorize the lyrics prior to their laboratory visit. Upon arrival at the laboratory, each participant provided written informed consent. Participants were then instructed to sing in unison with a partner's singing voice—either human or artificial—presented through wired earphones (MDR-EX155, Sony, Japan), and to follow the partner's singing as closely as possible (Figure 2). As in Nishiyama and Nonaka (2024), each trial began with two measures of metronome clicks to provide an initial tempo reference. Participants' singing voices were recorded using Cubase LE AI Elements 13 (Steinberg, Hamburg, Germany), with a Scarlett 2i2 Studio microphone, preamplifier, and USB audio interface (Focusrite, High Wycombe, UK). Before the experimental trials, participants completed several practice trials without visual information to familiarize themselves with the procedure and to confirm appropriate recording levels. The experiment comprised two partner conditions (human partner and artificial partner) and two visual conditions (with and without visual information). In the visual condition, participants viewed a video of the partner singing; in the no-visual condition, no video was presented. The display did not provide visible self-feedback for participants to monitor their own movements during the task. Participants completed five recording trials for each visual condition within each partner condition, resulting in a total of 20 trials per participant. To minimize learning or adaptation effects, the order of partner conditions was randomized within the experiment. To counterbalance order effects of visual information, six participants completed the visual condition first, whereas seven participants completed the no-visual condition first. Following the experimental session, participants completed a brief questionnaire assessing their ability to distinguish between the human and artificial partners and their subjective ease of singing. Responses varied across participants; notably, some participants reported difficulty distinguishing whether the singing voice or movements were human- or AI-generated, and several participants indicated that they were unaware that the movements shown in the artificial partner videos were AI-generated. Further details regarding the questionnaire are provided in the Supplementary material.

### 2.4 Data analysis

#### 2.4.1 Amplitude envelope

Vocal audio recordings were imported into MATLAB R2023b (MathWorks, Natick, MA) and resampled to 16 kHz. The temporal amplitude envelope of each vocal signal was extracted by computing the magnitude of the Hilbert transform (Issa et al., 2024; Braiman et al., 2018). Following prior work that examined temporal modulations in speech and music independently of spectral content (Ding et al., 2017; Elliott and Theunissen, 2009), slow fluctuations in sound amplitude were isolated by applying a third-order Butterworth infinite impulse response (IIR) low-pass filter with a cutoff frequency of 32 Hz. Filtering was performed in both forward and backward directions to avoid phase distortion. The resulting amplitude envelope time series were then down-sampled to 200 Hz, yielding a smooth representation of sound intensity fluctuations over time. The present envelope-based approach was adopted to enable comparison between human and synthesized singing voices while minimizing differences in phonetic characteristics inherent to each voice source. Approaches focusing on pitch, note accuracy, or discrete vocal onset timing may emphasize different aspects of joint singing performance, such as melodic precision or event synchronization. In contrast, amplitude envelope dynamics provide a broader representation of temporal fluctuations in vocal production and coordination that can be compared across acoustically different voice sources. This procedure produced amplitude envelope signals suitable for subsequent cross-correlation and Granger causality analyses, consistent with Nishiyama and Nonaka (2024).

#### 2.4.2 Cross correlation

As in Nishiyama and Nonaka (2024), similarity in temporal modulations of sound intensity between participants' singing and their partners' recorded voices was assessed using cross-correlation (CC) analysis. For each trial, CC functions were computed between the amplitude envelope time series of the partner's recording (human or Vocaloid) and the participant's singing voice while performing in unison. CC coefficients were calculated across the entire duration of each trial for time lags ranging from −0.1 to 0.1 s.

From the CC functions, three measures were extracted for each trial and participant. First, (1) the maximum CC value was defined as the highest correlation coefficient observed across all time lags and served as an index of overall similarity in amplitude envelope dynamics between the partner's and participant's voices. Second, (2) the lag at maximum correlation was identified as the time delay corresponding to the maximum CC value; positive lags indicate that the participant's voice lagged behind the partner's voice, whereas negative lags indicate that the participant's voice preceded the partner's voice. Third, (3) the zero-lag CC coefficient was computed by evaluating the CC function at zero lag, providing a measure of temporal synchrony or phase alignment between the amplitude envelopes of the partner's and participant's singing voices.

#### 2.4.3 Granger causality

Granger causality (GC) was computed between the amplitude envelope time series of the partner's singing voice (human or Vocaloid) and those of the participant's singing voice, in both directions, for each participant and each trial. Analyses were performed using the Multivariate Granger Causality (MVGC) Toolbox for MATLAB (Barnett and Seth, 2014), following the procedure described in Nishiyama and Nonaka (2024). For each trial and participant, the optimal model order—that is, the number of past time points included in the autoregressive model—was determined using the Akaike information criterion. To ensure consistency within participants, the maximum model order identified across the five trials within each visual condition was then applied to compute GC values for all five trials in that condition.

#### 2.4.4 Statistical analysis

The three cross-correlation (CC) measures—maximum CC, lag at maximum CC, and zero-lag CC—were analyzed using linear mixed-effects models implemented with the *lme* function from the *nlme* package (Pinheiro et al., 2024) in R (version 4.4.1). For each model, partner (human singer vs. artificial Vocaloid singer) and visual condition (Visual vs. No-Visual) were specified as fixed effects. Granger causality (GC) values were analyzed using a separate linear mixed-effects model with partner, visual condition, and direction of GC (from partner to participant vs. from participant to partner) specified as fixed effects. To account for the non-independence of repeated measurements, participant was included as a random intercept in all models. Post-hoc pairwise comparisons were conducted on estimated marginal means using the *emmeans* package (Lenth, 2024), with Bonferroni correction applied to control for multiple comparisons. Statistical significance was assessed at an alpha level of 0.05.

## 3 Results

**Figure 3:**
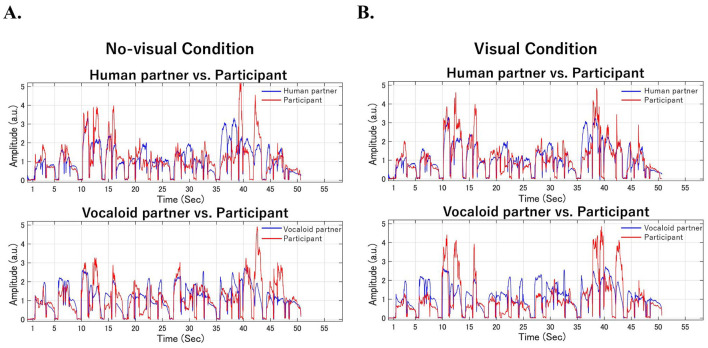
Representative examples of amplitude envelope time series of singing voices for the two partner conditions (human partner and an artificial Vocaloid partner) under **(A)** the No-Visual condition and **(B)** the Visual condition from the experiment. Examples are taken from the fifth trial of participant P5.

Figure 3 presents examples of amplitude envelope time series of singing voices for the two partner conditions (human partner and an artificial Vocaloid partner) under (A) the No-Visual condition and (B) the Visual condition from the experiment.

**Figure 4:**
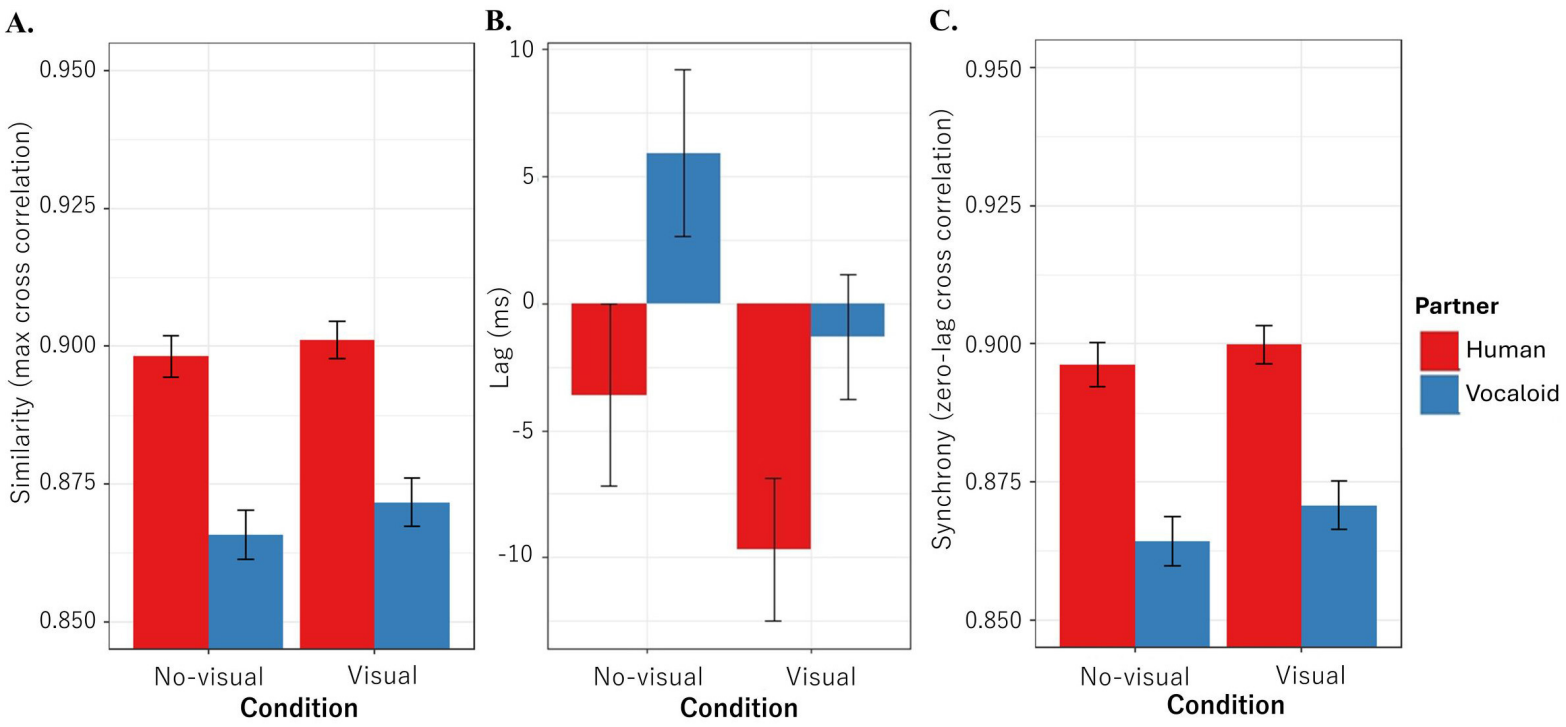
The results of cross correlation (CC) analysis of the temporal modulations in sound amplitude between the partner's and the participant's singing voices as a function of visual condition (No-Visual vs. Visual) and partner type (Human or Vocaloid). **(A)** Maximum CC coefficients across all time lags, indexing overall similarity in amplitude envelope dynamics between the partner and participant. **(B)** Time lag at which the maximum CC occurred, indicating the relative temporal offset between the two time series that yielded the highest similarity; positive values indicate that the participant's singing voice lagged behind the partner's voice, whereas negative values indicate that the participant's voice preceded the partner's voice. **(C)** Zero-lag CC coefficients, indexing phase alignment (synchrony) between the amplitude envelopes of the partner's and participant's singing voices. Error bars represent ±1 standard error of the mean.

Across participants, singing with a human partner exhibited greater degree of similarity to the temporal modulation of sound intensity with the human partner compared to the artificial Vocaloid partner (Figure 4). Pooling all trials, the cross-correlation functions showed higher peaks in the human-partner condition, indicating greater overall similarity in amplitude-envelope dynamics. A linear mixed-effects model on maximum CC confirmed a robust main effect of partner, *F*_(1, 244)_ = 114.18, *p* < 0.0001, with no other significant effects. Thus, the temporal modulation patterns of participants' voices were more similar to the human partner's voice than to the Vocaloid partner's voice, regardless of visual condition.

The lag at maximum CC revealed a systematic difference in temporal alignment between partner types (Figure 4B). With the human partner, participants tended to sing *ahead* of the partner (negative lags), consistent with anticipatory coordination. This anticipatory tendency was stronger when visual information was available. In contrast, in the No-Visual condition with the Vocaloid partner, participants tended to lag behind the partner (positive lags), and in the Visual condition they shifted toward smaller lags but still showed comparatively less anticipation than in the human-partner condition. Mixed-effects modeling confirmed main effects of partner, *F*_(1, 244)_ = 32.14, *p* < 0.0001, and visual condition, *F*_(1, 244)_ = 17.72, *p* < 0.0001, indicating that participants generally sang further ahead of their partner when visual information was present. No other effects were significant.

A similar pattern emerged for zero-lag CC, an index of synchrony when the two time series are aligned in time (Figure 4C). Participants showed higher zero-lag correlations with the human partner than with the Vocaloid partner. The mixed-effects model revealed a significant main effect of partner, *F*_(1, 244)_ = 108.47, *p* < 0.0001, with no other significant effects. Together, the CC analyses converge on two key results: (1) vocal dynamics were more tightly coupled to the human partner than to the artificial partner (similarity and synchrony), and (2) the temporal relation shifted toward greater anticipation when visual information was available, particularly in the human-partner condition.

**Figure 5:**
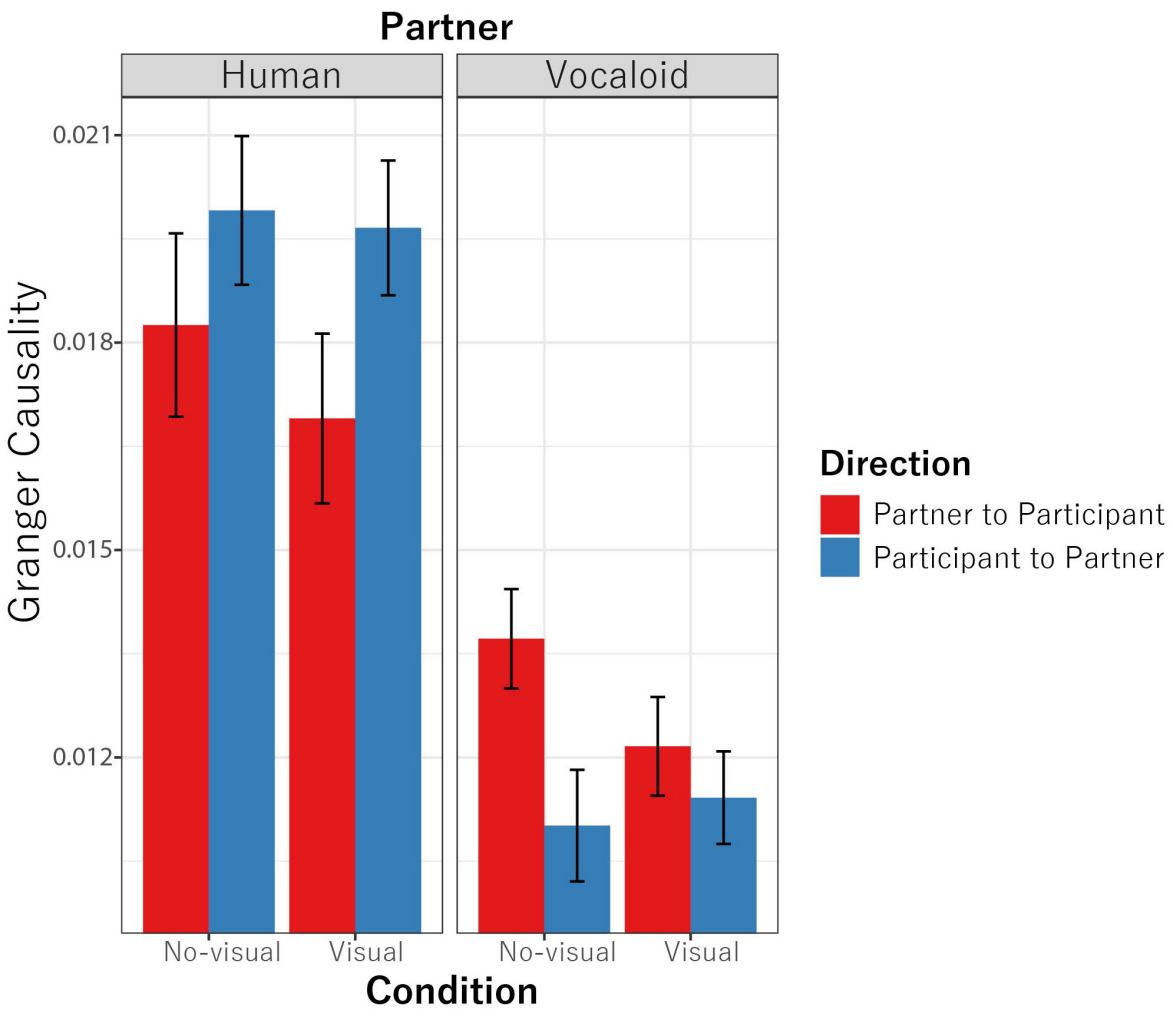
Results of Granger causality (GC) analysis of temporal modulations in sound amplitude between the partner's and participant's singing voices as a function of visual condition (No-Visual vs. Visual) and partner type (Human or Vocaloid). Granger causality values are shown for both directions of influence, from the partner's vocal recording to the participant's performance and from the participant's performance to the partner's vocal recording. Error bars represent ±1 standard error of the mean.

Figure 5 shows Granger-causality (GC) magnitudes in both directions (from partner to participant and from participant to partner) for each visual condition. As expected for a fixed recording, trials with the Vocaloid partner generally showed a pattern consistent with participants following the partner. In contrast, trials with the human partner showed the reverse tendency: participants' dynamics more strongly anticipated the partner's dynamics, consistent with anticipatory coordination. A linear mixed-effects model confirmed a significant main effect of partner, *F*_(1, 500)_ = 101.86, *p* < 0.0001, and a significant partner × direction interaction, *F*_(1, 500)_ = 9.02, *p* = 0.0028. Post-hoc comparisons indicated higher GC values *f* or the human partner than for the Vocaloid partner in both directions and both visual conditions, with the difference especially pronounced in the participant-to-partner direction. In the No-Visual condition, GC values were greater for the human partner than for the Vocaloid partner in the partner-to-participant direction (estimate = 0.00454, SE = 0.00131, *p* = 0.0006) and the participant-to-partner direction (estimate = 0.00890, SE = 0.00131, *p* < 0.0001). The Visual condition showed the same pattern (partner to participant: estimate = 0.00474, SE = 0.00131, *p* = 0.0003; participant to partner: estimate = 0.00824, SE = 0.00131, *p* < 0.0001). No additional effects were significant. Thus, GC analyses reinforce the CC findings: interaction with a human partner was characterized by stronger anticipatory structure and a directional asymmetry consistent with anticipatory coordination.

**Figure 6:**
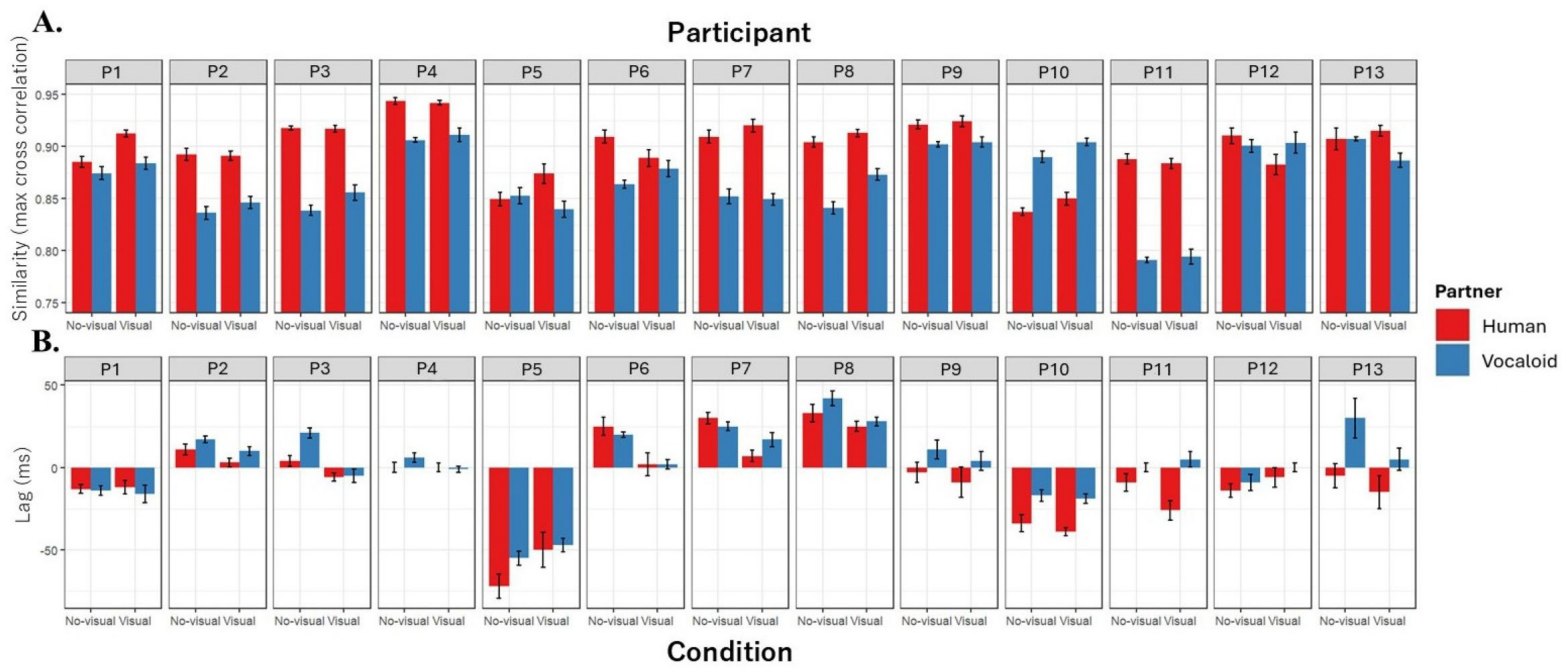
Individual-participant results of the cross-correlation (CC) analysis of temporal modulations in sound amplitude between the partner's and participant's singing voices as a function of visual condition (No-Visual vs. Visual) and partner type (Human vs. Vocaloid). Panels show **(A)** maximum CC coefficients and **(B)** the time lag at which the maximum correlation occurred for each participant (P1–P13).

**Figure 7:**
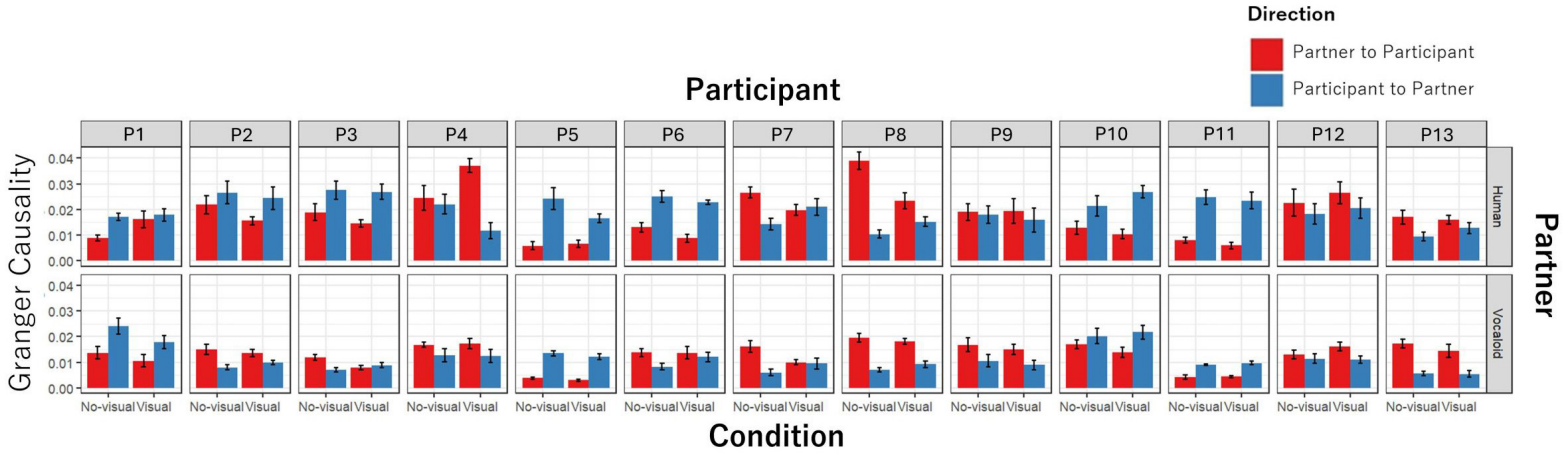
Individual-participant results of the Granger causality (GC) analysis of temporal modulations in sound amplitude between the partner's and participant's singing voices as a function of visual condition (No-Visual vs. Visual) and partner type (Human vs. Vocaloid). GC values are shown for both directions of influence, from the partner's vocal recording to the participant's performance and from the participant's performance to the partner's vocal recording, for Participants P1–P13. Error bars represent ±1 standard error of the mean.

At the individual level, most participants showed greater similarity in amplitude-envelope dynamics with the human partner than with the Vocaloid partner in both visual conditions (Figure 6), with only a small number of exceptions. GC profiles also revealed stable individual strategies across conditions: some participants tended to follow the partner, whereas others tended to lead (Figure 7). Despite this variability, the overall pattern was consistent: participants exhibited stronger anticipatory coordination with the human partner than with the artificial partner across both visual conditions.

## 4 Discussion

This study investigated how coordination dynamics in joint singing differ between human–human and human–artificial partner interactions, and how visual information modulates these dynamics. Extending Nishiyama and Nonaka (2024), we introduced visual access to the partner's movements—either recorded human movement or AI-generated movement synchronized to synthesized singing—to test whether visual information could close the coordination gap between human–human and human–machine joint singing.

Across measures, singing with a human partner produced stronger coupling than singing with an artificial partner. Maximum CC and zero-lag CC showed that participants' vocal dynamics were more similar to, and more synchronous with, the human partner than the Vocaloid partner. Critically, temporal alignment measures pointed to a qualitative difference in coordination mode: with the human partner, participants tended to sing ahead of the partner (negative lags), whereas with the Vocaloid partner they tended to lag behind, especially without visual information. GC results converged on the same conclusion. Although both partner conditions necessarily involved prospective control (participants had to align to a partner recording), human–human singing exhibited stronger prospectivity and a directional asymmetry consistent with anticipatory coordination. These results suggest that the “being-with” of singing is not captured solely by stable tempo or a superficially human-like voice. Instead, human–human coordination appears to depend on subtle, time-varying structure in vocal production that supports prospective control and timing adjustment.

Adding visual information shifted coordination toward greater anticipation, as evidenced by the main effect of visual condition on lag at maximum CC. This aligns with prior work showing that body movement provides information that complements audition for anticipating others' timing and actions (Keller et al., 2007; Burger et al., 2014; Sabharwal et al., 2024). Importantly, however, visual information did not erase the qualitative difference between partner types. Even when participants could see an AI-generated partner body that moved in synchrony with the synthesized voice, human–human singing still exhibited stronger coupling and more pronounced anticipatory dynamics.

One implication is that vision enhances coordination by increasing the availability of prospective information. Human singing is produced by coupled bodily processes (respiration, laryngeal biomechanics, articulation) that generate lawful micro-variations in both sound and movement. These micro-variations may serve as prospective information—auditory (e.g., inhalation) and visual (e.g., subtle upper-body motion preceding phrase onset)—that are difficult to reproduce convincingly in synthesized audio and AI-generated motion. This interpretation aligns with qualitative studies of musical interaction, which suggest that mutual visibility supports coordination by enabling performers to perceive prospective bodily information and anticipate upcoming sounds and actions (Pentimalli and Gobo, 2023). Even high-quality artificial partners may provide timing information that is “good enough” to support basic alignment, while still lacking the fine-grained structure that supports strong anticipatory synchronization.

A related factor concerns tempo variability. Whereas the artificial partner maintained a constant tempo, the human partner exhibited natural tempo fluctuations, and these fluctuations likely to contributed to the enhanced anticipatory synchrony observed in human-human singing. This result is consistent with the view that interpersonal coordination relies on adaptive coupling and predictive timing processes that respond to temporal fluctuations in a partner's behavior (Repp and Su, 2013). Crucially, such variability is characteristic of human performance: subtle fluctuations in tempo and amplitude arise from biomechanical and expressive constraints inherent in vocal production. The artificial partner in the present study, by contrast, was generated with a standardized temporal structure, producing a signal that was more isochronous and exhibited fewer dynamic fluctuations. This reduction in natural variability may have limited the availability of the fine-grained prospective information that underpins anticipatory synchronization.

The reduced similarity (maximum CC) and synchrony (zero-lag CC) observed with Vocaloid may reflect acoustic properties that are difficult to reproduce without the human body. Although the present envelope-based analysis was designed to minimize phonetic differences between synthesized and human voices, machine-specific acoustic regularities may nonetheless have contributed to the observed coordination patterns. Even when pitch trajectories are closely matched, synthesized voices may lack the fine dynamic fluctuations inherent to human vocal production (Tamura et al., 2015)—fluctuations that are likely reflected in amplitude-envelope structure. Because two human singers share common biomechanical constraints, their envelope dynamics may naturally fall within a similar “family” of temporal patterns, making close coupling easier to achieve. The Vocaloid voice, by contrast, may carry machine-specific regularities or artifacts that persist even after filtering and envelope extraction, thereby reducing similarity and limiting the emergence of human-like coordination dynamics.

Although individual participants differed in their overall tendency to lead or follow, the human–human advantage was robust across participants and conditions. This pattern indicates that the observed effects are not driven by a single coordination strategy, but reflect a general shift in the quality of coupling when the partner is human. In other words, participants may adopt different global timing policies, yet still exploit human-specific auditory–visual structure to achieve stronger anticipatory synchronization with a human partner.

Several limitations of the present study should be acknowledged. Firstly, the sample size was relatively small, which may have limited the statistical power and generalizability of the findings. Although mixed-effects models partially mitigate this issue by accounting for within-participant variability, future studies should examine a larger and more diverse sample population. Despite these limitations, the present findings consistently point to qualitative differences in the coordination dynamics established with human vs. artificial partners.

These findings may have implications for the design of artificial partners intended to support human-like social and musical interaction. Improving realism may require more than refining timbre or adding visually plausible motion; it may require reproducing the micro-structure of human vocal–bodily dynamics that provides prospective information for coordination. In the present study, the visual stimulus of the artificial partner was generated from animation based on a static image, which may have lacked subtle biological motion cues that support anticipatory synchronization. Such cues may include respiration-related movement, pre-phonatory bodily motion, and fine-grained timing fluctuations that emerge from coupled vocal–motor processes. The extent to which these bodily cues contribute to anticipatory synchronization remains to be clarified. Future work should therefore examine coordination not only in the vocal signal but also in bodily movement during singing, including respiration-related motion and postural sway. In parallel, candidate sources of information—for example, breath sounds, pre-phonatory motion—should be experimentally manipulated to determine which information most strongly supports anticipatory synchronization. Once identified, such information could be systematically implemented in voice synthesis systems and embodied agents to test whether doing so brings human–machine interaction closer to the dynamics of human–human musical coordination.

## 5 Conclusion

The present study examined how coordination dynamics in singing differ when a human singer performs with a human partner vs. an artificial partner generated using VOCALOID6, and whether the availability of visual information modulates these dynamics. Using cross-correlation and Granger causality analyses of vocal amplitude envelopes, we assessed similarity, synchrony, and anticipatory relationships between participant's singing voice and that of the partner.

Across all measures, singing with a human partner elicited stronger coupling than singing with an artificial partner. Participants showed greater similarity and synchrony in vocal dynamics with the human partner, as well as more pronounced anticipatory synchronization—indicating a shift from reactive alignment toward anticipatory coordination. The availability of visual information further enhanced anticipatory timing but did not eliminate the qualitative difference between human–human and human–machine interaction. Even when provided with AI-generated visual movement synchronized to synthesized singing, coordination with an artificial partner did not reach the level observed with a human partner.

These findings suggest that effective interpersonal coordination in singing depends on subtle, time-varying structure in both vocal and bodily dynamics—structure that current voice synthesis and animation technologies may not yet fully capture. This structure appears to provide rich prospective information that supports anticipation and mutual adjustment during joint performance. More broadly, musical interaction offers a sensitive model system for probing the limits of human–machine coordination and for identifying the informational and biomechanical constraints that underlie genuinely human-like social interaction.

## Data Availability

The raw data supporting the conclusions of this article will be made available by the authors, without undue reservation.
