# Supplementary material for: Coordination dynamics in singing with human and artificial partners: the role of visual information
Source: Front Cognit. 2026 Jun 15;5:1810330. doi: 10.3389/fcogn.2026.1810330 (PMC13281077; doi:10.3389/fcogn.2026.1810330)
Supplement: Supplementary file 1 [file Data_Sheet_1.pdf]

Coordination Dynamics in Singing with Human and Artificial Partners: The Role of Visual Information

Rina Nishiyama[1]*, Tetsushi Nonaka[1]

[1]Graduate School of Human Development and Environment, Kobe University, Kobe, Japan

*** Correspondence:**
Rina Nishiyama
nsymrn271911@gmail.com

Description of Additional Supplementary Files

Supplementary Movie S1 | Video of the human partner singing Silent Night in Japanese, used in the human partner condition with visual information.

URL: https://youtu.be/TmZ8_SdgOa4

Supplementary Movie S2 | Video of the artificial partner generated from a synthesized singing voice using an AI video platform (Hedra Character 3), used in the artificial partner condition with visual information.
URL: https://youtu.be/88zgoKHFbvU

Supplementary Audio S3 | Audio of the human partner singing Silent Night in Japanese, used in the human partner condition.

URL: https://drive.google.com/file/d/1nuz8C_BC4j_Gz9KnSTz7-_YYl50eJYLj/view?usp=sharing

Supplementary Audio S4 | Audio of the artificial partner generated from a synthesized singing voice using VOCALOID AI (voice: HARUKA, VOCALOID 6 voice synthesis software), used in the artificial partner condition.
URL:
https://drive.google.com/file/d/1Auqd4bBCrDQUQSFM6DvJY7HoRWouY2lP/view?usp=sharing

This is a provisional file, not the final typeset article
